# Shared gut microbiota features between children with autism spectrum disorder and their mothers

**DOI:** 10.3389/fmed.2026.1873159

**Published:** 2026-09-09

**Authors:** Fangtao Xiang, Xirong Wan, Xuebing Han, Tao Zhuang, Hanyu Wang

**Affiliations:** 1 Sichuan Provincial Key Laboratory of Philosophy and Social Sciences for Language Intelligence in Special Education, Leshan Normal University, Leshan, China; 2 Leshan Central District Maternal and Child Health Hospital, Leshan, China; 3 College of New Energy Materials and Chemistry, Leshan Normal University, Leshan, China; 4 Guanghan Maternal and Child Health Hospital, Guanghan, China

**Keywords:** 16S rRNA gene sequencing, autism spectrum disorder, gut microbiota, mother–child pair, vertical transmission

## Abstract

**Objective:**

To explore shared gut microbiota and short-chain fatty acid (SCFA) features between children with autism spectrum disorder (ASD) and their mothers, and to identify key taxa potentially influencing ASD via vertical transmission.

**Methods:**

Thirty seven mother–child dyads with ASD were enrolled. Fecal samples were analyzed by 16S rRNA gene sequencing and targeted SCFA metabolomics.

**Results:**

The average mother–child shared ASV proportion was 23.92% (relative to child total). Fifteen genera showed significant positive correlations between mothers and children after FDR correction (*P* < 0.05), including Faecalibacterium (r = 0.518), Subdoligranulum (r = 0.462), and Roseburia hominis (r = 0.458). Propionic acid also exhibited a significant positive correlation (r = 0.418, *P* = 0.024). Stratified analysis showed that vaginal delivery was associated with significantly higher sharing than Cesarean section. External validation in two public datasets identified Intestinibacter as a consistently ASD-enriched genus.

**Conclusion:**

There is moderate gut microbiota overlap between ASD children and their mothers. Fifteen genera and propionic acid may be vertically transmitted and contribute to ASD, offering potential targets for early intervention.

## Introduction

Autism spectrum disorder (ASD) is a complex, common, and multifactorial neurodevelopmental disorder with childhood onset. It is characterized by deficits in communication and social interaction, as well as repetitive behaviors and restricted interests (1, 2). According to the 2021 Global Burden of Disease Study, ASD affects approximately 61.8 million people worldwide (3). Over the past two decades, the prevalence of ASD among 8-year-old children has risen significantly, from 6.7 per 1,000 children (1 in 150) in 2000 to 32.2 per 1,000 (1 in 31) in 2022 (4). The prevalence of ASD is more than four times higher in boys than in girls. It is often accompanied by comorbid conditions such as epilepsy, depression, anxiety, and attention-deficit/hyperactivity disorder, as well as challenging behaviors including sleep problems and self-injury (5, 6). In Asia, the average prevalence of ASD increased from approximately 1.9 per 10,000 before 1980 to 14.8 per 10,000 between 1980 and 2010 (7). Studies published between 1996 and 2001, covering the United Kingdom, the United States, Scandinavia, and Japan, reported that prevalence might range from 30 to 60 per 10,000 (8). Although these epidemiological features are well understood, ASD shows strong clinical heterogeneity, and its underlying causes and pathogenesis remain further elucidated.

From a phenotypic perspective, ASD is a heterogeneous neurodevelopmental disorder. Its core symptoms include altered social interaction, communication deficits, and restricted, repetitive behaviors (including sensory sensitivity) (9). Beyond these core symptoms, individuals with ASD may also experience challenges in emotion regulation, cognition, and adaptive functioning. These challenges range from intellectual disability (requiring high support needs) to high cognitive skills (requiring lower support needs) (10, 11). Although the exact etiology of the disorder remains unclear, the diverse clinical manifestations of ASD result from complex interactions among genetic, neurobiological, and environmental factors (12). A large population-based study covering five countries, which included more than 20,000 ASD patients, estimated the heritability of ASD at approximately 80% (median heritability 80.8%; 95% CI, 73.2%–85.5%). For the Nordic countries specifically, heritability estimates ranged from 81.2% to 82.7% (13). This suggests that most of the variation in ASD traits arises from genetic factors, with no support for contribution from maternal effects. However, environmental factors also play a substantial role, with estimates suggesting they may contribute up to 40%–50% of the variance in ASD risk (14). Existing evidence also indicates multiple prenatal and perinatal environmental risk factors for ASD. These include advanced parental age, maternal health conditions, birth complications, and prenatal chemical exposure (15). These environmental risk factors may interact with genetic predispositions to alter neurodevelopmental trajectories through various mechanisms. Such mechanisms include immune dysregulation, mitochondrial dysfunction, hormonal disorders, gut microbiome alterations, and oxidative stress (16–18). Among these environmental influences, the gut microbiota, particularly mother-to-child microbial transmission during early life, has emerged as a critical modifiable factor. However, the role of postnatal mother-child microbial sharing in ASD remains underexplored.

In recent years, a growing number of studies have highlighted the importance of maternal gut microbiota composition and fatty acid metabolism during pregnancy in the pathogenesis of ASD (18). Existing evidence shows that maternal gut dysbiosis can regulate fetal neurodevelopment through multiple transgenerational mechanisms, such as metabolite-mediated pathways, immune pathway activation, and epigenetic reprogramming. This significantly increases the risk of ASD in offspring (19). Using amplicon sequence variant technology, Chen and colleagues analyzed 76 children with ASD and 47 age-and sex-matched typically developing children together with their mothers. They found that gut bacteria shared by children with ASD and their mothers were significantly associated with the children's developmental levels and the severity of social deficits. This suggests that these shared microbiota may play an important role in the development of ASD (20). Moreover, maternal fatty acid metabolism disorders have also been closely linked to ASD risk in offspring. Studies have found that a lower maternal *ω*-3/*ω*-6 polyunsaturated fatty acid ratio and docosahexaenoic acid deficiency during pregnancy are associated with a higher risk of autistic traits or ASD with intellectual disability in offspring. In addition, cord blood and neonatal lipidomic analyses have repeatedly observed disturbances in fatty acid metabolism, mitochondrial *β*-oxidation, and inflammatory lipid mediators (21). These findings suggest that ASD patients and their mothers may share common gut microbial features and fatty acid metabolic abnormalities. Such shared features could influence the development of the disorder through mother-child biological associations.

This study aimed to evaluate the associations of shared gut microbes and fatty acid profiles between ASD children and their mothers with the children's clinical scores (CARS). To this end, we will use targeted metabolomics to analyze fatty acids and their related metabolites, combined with 16S rRNA gene amplicon sequencing for compositional and diversity analysis of fecal samples. We hope to reveal microbe-metabolite features shared by mothers and children and their potential value in assessing ASD severity.

## Methods

### 2.1 Clinical sample collection

This study recruited 37 children with ASD and their mothers. All participants were patients from pediatric clinics and community settings. Each enrolled child was screened using the Childhood Autism Rating Scale (CARS score) and subsequently diagnosed with autism spectrum disorder by Leshan Central District Maternal and Child Health Hospital. Exclusion criteria included ASD children with other neurological disorders (e.g., epilepsy) or severe organic diseases (e.g., liver dysfunction). All participants provided written informed consent. The study protocol was approved by Leshan Normal University. Demographic and clinical information for both children and mothers was collected using standardized questionnaires, including child age, gender, disease duration, CARS score, delivery mode, feeding history (breastfeeding/mixed feeding/formula feeding), defecation frequency, antibiotic use within the past 3 months, as well as maternal age at delivery, residence (urban/suburban/rural), and pet ownership.

We confirmed that none of the children had received antibiotics within one month prior to fecal sample collection. Fecal samples were first collected from the children. Maternal samples were collected the next day. All samples were collected in fecal collection tubes containing a DNA stabilizer. The mothers completed the collection at home. These tubes preserve the integrity of gut bacteria in feces for at least 24 h at room temperature. After collection, the samples were immediately transferred to OE Biotech Co., Ltd. institution and stored at −80 °C until DNA extraction.

### 2.2 Fecal DNA extraction and 16S rRNA gene V3-V4 region sequencing

Fecal microbial DNA was extracted using the MagPureSoil DNA LQ Kit (Magan). The concentration and purity of DNA were measured with a NanoDrop 2000 (Thermo Fisher Scientific, USA). Agarose gel electrophoresis was also used to assess DNA purity. Then, the V3-V4 region of the 16S rRNA gene was amplified. The amplification used barcode-specific primers and Takara ExTaq high-fidelity enzyme. The primer sequences were 343F (5'-TACGGRAGGCAGCAG-3’) and 798R (5'-AGGGTATCTAATCCT-3’). The amplification involved two steps. The first step amplified the V3-V4 region. The second step was an index PCR. The amplified products were sequenced on the Illumina NovaSeq 6000 platform. This generated 250 bp paired-end reads. The sequencing work was completed by Shanghai OE Biotech Co., Ltd. (Shanghai, China).

### 2.3 Bioinformatics analysis of sequencing data

OE Biotech Co., Ltd. (Shanghai, China)performed the library construction, sequencing, and data analysis for this study. The raw FASTQ data were processed with cutadapt software. This step removed primer sequences. Then, quality control was performed using the DADA2 method within the QIIME2 (2020.11) platform. Default parameters were used. The QC steps included quality filtering, denoising, read merging, and chimera removal. After these steps, representative sequences and an ASV abundance table were obtained. The representative sequences of each ASV were extracted using the QIIME2 software package. These sequences were then aligned and annotated against reference databases. The Silva database (version 138) was used for 16S rRNA gene sequence alignment. Species annotation was performed with the q2-feature-classifier software. All parameters were set to their default values.

### 2.4 Short-chain fatty acid (SCFA) analysis

SCFA analysis was performed as described previously (22), with the following brief procedure. Fecal samples were homogenized with ultrapure water at a ratio of 1:10 (w/v). The homogenate was then acidified with 1% formic acid. After acidification, the sample was centrifuged at 12,000×g for 10 min at 4 °C. The supernatant was filtered through a 0.22-*μ*m filter. Internal standards (^2^H₄-acetate and ^13^C₃-propionate) were added. SCFA quantification was carried out on a gas chromatograph-mass spectrometer (Agilent 7890B/5977A) equipped with a DB-FFAP capillary column (30 m × 0.25 mm). The column temperature was programmed as follows: initial temperature 50 °C held for 2 min, then increased to 230 °C at a rate of 10 °C/min. A calibration curve was established using mixed standards (Sigma-Aldrich), with a coefficient of determination R^2^ > 0.99. Final data were normalized per gram of wet feces. The Mann-Whitney U test was used for group comparisons, and the significance level was set at *P* < 0.05.

### 2.5 External validation using public datasets

Since no healthy control group was included in this study, we were unable to directly assess whether the core microbial markers shared between mothers and children are ASD-specific. To independently validate the association of key genera identified in our study with ASD phenotype, we incorporated two public datasets (PRJNA453621, PRJNA1018910) for external differential abundance analysis. Differential abundance analyses were performed using the Wilcoxon rank-sum test, with *P* < 0.05 considered statistically significant. Detailed data retrieval, quality control, and processing procedures are described in Supplementary Methods.

### 2.6 Statistical analysis

All statistical analyses were performed using SPSS 22.0 and R software (version 4.1.0). All continuous variables were first assessed for normality using the Shapiro–Wilk test. The results indicated that these variables (e.g., gut microbiota relative abundances and CARS scores) did not follow a normal distribution (*p* < 0.05). Therefore, non-parametric methods were applied: the Mann–Whitney U test was used for between-group comparisons of continuous variables, and Spearman's rank correlation coefficient was used to evaluate associations among bacterial genus abundances, fatty acid levels, and the CARS score. Categorical variables were analyzed using the chi-square test. Bray-Curtis distances were calculated to assess the similarity of gut microbiota composition between mother-child pairs. To control the false discovery rate for multiple comparisons, the Benjamini-Hochberg FDR correction was applied, and an adjusted *P* < 0.05 was considered statistically significant. A two-sided *P* < 0.05 was considered statistically significant. Continuous variables are presented as mean ± standard deviation (SD) with range; categorical variables are presented as counts (percentages).

## Result

### 3.1 Baseline characteristics

A total of 37 children with ASD (29 male, 8 female; mean age 6.59 ± 4.14 years, range 1.3–17.2) and their mothers were enrolled. The mean CARS score was 43.1 ± 5.57 (range 32.0–54.3), indicating moderate-to-severe autism symptoms. The mean age at ASD diagnosis was 4.03 ± 3.04 years, and the mean disease duration was 3.85 ± 2.69 years. Among the enrolled children, 22 (59.5%) presented with gastrointestinal symptoms. Regarding delivery mode, 14 pairs (37.8%) were vaginal deliveries and 23 pairs (62.2%) were Cesarean sections. Detailed demographic and clinical characteristics are summarized in Table 1.

**Table 1:** Demographic and clinical characteristics of ASD mother-child dyads (*N* = 37).

Child characteristics	Value
Age (years), mean ± SD (range)	6.6 ± 4.1 (1–17)
Gender	
Male	29 (78.4%)
Female	8 (21.6%)
Age at ASD diagnosis (years), mean ± SD (range)	4.0 ± 3.0 (1.0–16.0)
Disease duration (years), mean ± SD (range)	3.9 ± 2.7 (1.0–11.0)
CARS score, mean ± SD (range)	43.1 ± 5.57 (32.0–54.3)
Delivery mode	
Vaginal	16 (43.2%)
Cesarean	21 (56.8%)
Gastrointestinal symptoms	
Yes	22 (59.5%)
No	15 (40.5%)
Feeding history	
Breast	15 (40.5%)
Mixed	7 (18.9%)
Formula	15 (40.5%)
Child dietary preferences (multiple choices)	
High-fiber (vegetables/whole grains)	22 (59.5%)
Dairy products	17 (45.9%)
Processed foods	5 (13.5%)
Fermented foods (yogurt/kimchi)	8 (21.6%)
Vegetarian-predominant	10 (27.0%)
Others	7 (18.9%)
Child defecation frequency	
Once daily	28 (75.7%)
Twice daily	9 (24.3%)
Child antibiotic use (past 3 months)	
Yes	10 (27.0%)
No	27 (73.0%)
Child age at stool collection (years), mean ± SD (range)	6.6 ± 4.1 (1–17)
Maternal characteristics	
Maternal age at delivery (years), mean ± SD (range)	33.6 ± 6.2 (22.0–46.0)
Maternal age at stool collection (years), mean ± SD (range)	36.8 ± 5.2（28–53）
Residence	
Urban	22 (59.5%)
Suburban	11 (29.7%)
Rural	4 (10.8%)
Pet ownership	
Yes	6 (16.2%)
No	31 (83.8%)
Informed consent	
Consent for study participation (Yes)	37 (100%)
Consent for anonymized data publication (Yes)	37 (100%)

### 3.2 Quality control of 16S sequencing and SCFA data

**Figure 1:**
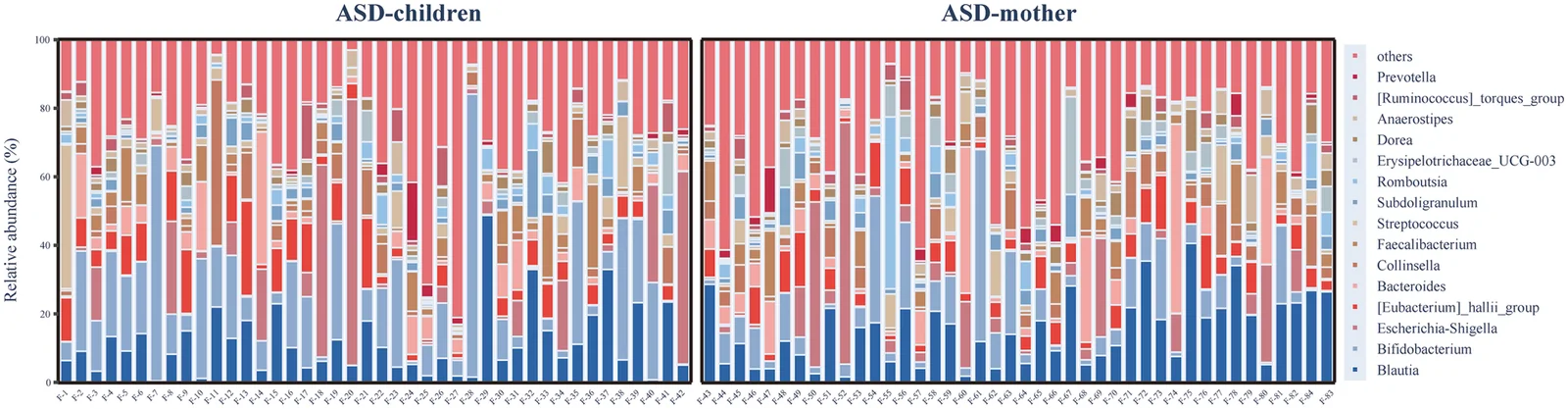
Bar chart of community structure of the top 15 genera at the genus level. Each bar represents a sample, and different colors represent different genera. Others represents all genera other than the top 15.

In this study, 74 fecal samples (from 37 mother-child pairs) were subjected to 16S rRNA gene V3-V4 region sequencing. After quality control, denoising, assembly, and chimera removal of the raw 16S sequencing data, a total of 5,323,202 valid sequences were obtained, with an average of 63,371 sequences per sample (see Supplementary Table S1). The bar chart (Figure 1) displays the top 15 genera in terms of abundance at the genus level.

Additionally, we used a gas chromatography-mass spectrometry system to detect six major SCFAs (acetic acid, propionic acid, butyric acid, isobutyric acid, valeric acid, and isovaleric acid) in 74 samples (from 37 mother-child pairs). The coefficients of determination (R^2^) of the calibration curves established with mixed standards were all greater than 0.99. Among these, acetic acid, propionic acid, and butyric acid were the major components, accounting for more than 90% of the total SCFAs. After quality control to remove abnormal or missing data, valid data for acetic acid, propionic acid, and butyric acid were obtained from 74 samples (from 37 mother-child pairs) (Supplementary Table S2). Subsequent analyses were performed using the 16S rRNA sequencing data of the 37 mother-child pairs that matched these 74 samples.

### 3.3 Analysis of shared gut microbiota features between ASD children and their mothers

**Figure 2:**
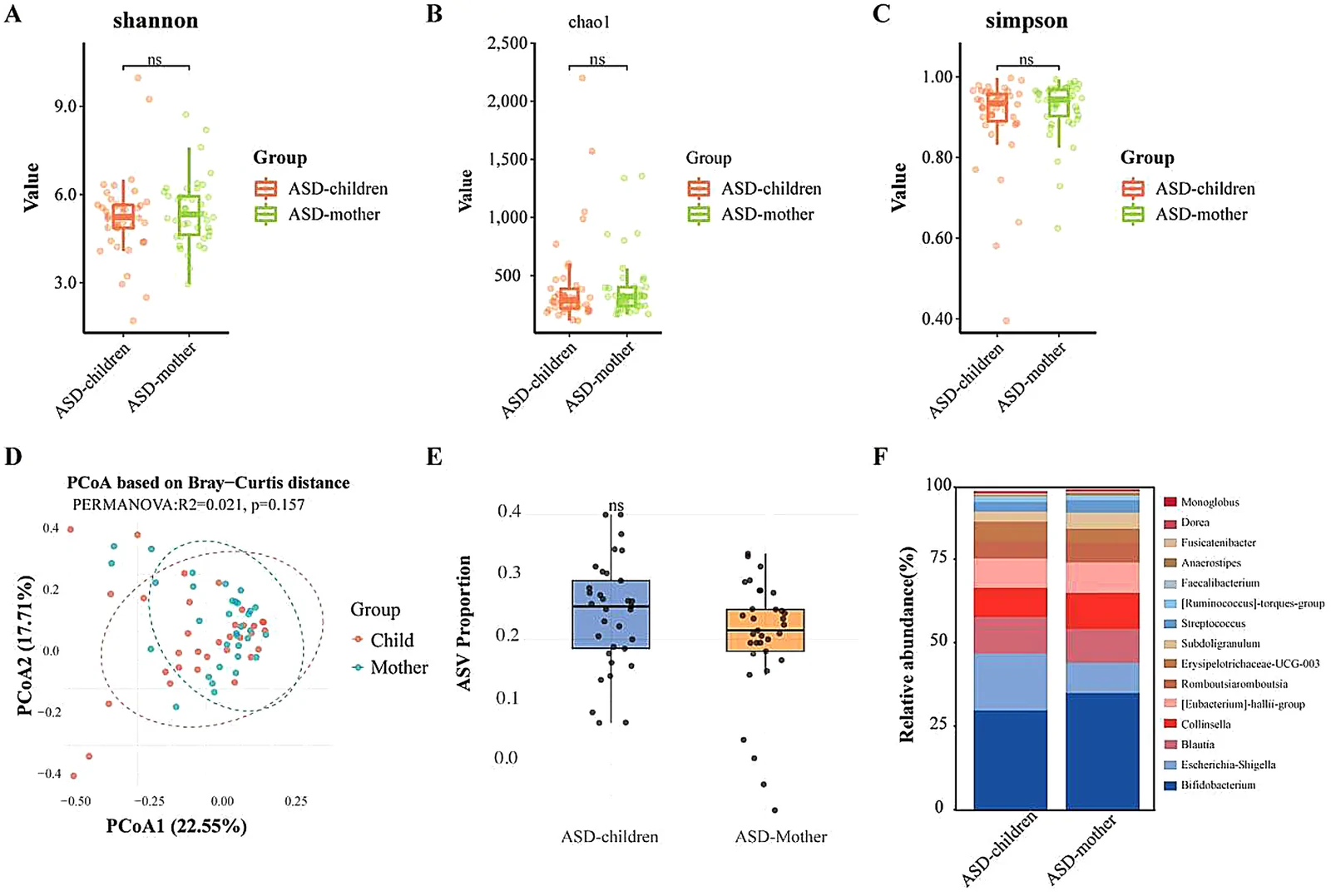
Shared gut microbiota characteristics between ASD children and their mothers. A–C: Boxplots showing the comparison of alpha diversity indices between children and mothers **(A)** Shannon index, **(B)** Chao1 index, and **(C)** Simpson index. No significant differences were observed (Mann-Whitney U test, *P* > 0.05); **(D)** PCoA plot based on Bray-Curtis distances, illustrating the overall gut microbial community composition of children and mothers; **(E)** Boxplot comparing the number of ASVs between children and mothers; **(F)** Bar chart showing the top 15 dominant genera based on average relative abundance of ASVs shared between mother-child pairs.

To explore the relationship between gut microbiota features of ASD children and their mothers, we first compared alpha diversity between mother-child pairs. Shannon index (measuring richness and evenness), Chao1 index (measuring richness), and Simpson index were used to assess similarity in species diversity. The Mann–Whitney U test was applied for comparison. Boxplots (Figures 2A–C) showed no significant differences in any alpha diversity index between children and mothers (*P* > 0.05).

Next, we quantified the similarity of gut microbiota composition using Bray–Curtis distance and visualized it with principal coordinate analysis (PCoA). As shown in Figure 2D, the first two principal coordinates (PCoA1 and PCoA2) explained 22.55% and 17.71% of the community variation, respectively. In the two-dimensional plot, sample points and confidence ellipses of children and mothers largely overlapped. PERMANOVA further confirmed no significant difference in overall microbial community composition between the two groups (R^2^ = 0.021, *P* = 0.157).

Finally, we counted the number of ASVs for each child and each mother (Supplementary Table S3). The average ASV count was 103.70 ± 64.13 (median 91 [16, 293]) for children and 114.84 ± 67.22 (median 102[23, 306]) for mothers. Figure 2E shows boxplots of ASV number distributions. On average, mother-child pairs shared 24.81 ASVs (median=22.0 [16, 31]). The average shared proportion was 23.92% relative to child total ASVs, 21.60% relative to mother total ASVs, with a mean Jaccard similarity index (shared ASVs/union ASVs) of 12.80%. To determine whether the observed sharing exceeded random expectation, we performed a permutation test (randomly permuting mother-child pairings 999 times). The observed mean of 24.81 shared ASVs was significantly higher than the null expectation (*P* < 0.001), indicating that the observed sharing was not driven by random chance. These findings indicate a moderate degree of gut microbiota overlap between ASD mothers and their children.

To identify key shared gut bacteria linked to ASD, we focused on ASVs common to both mothers and children. Based on their average relative abundance, we selected the top 50 dominant genera (Supplementary Table S4). The top 15 of these are shown in Figure 2F. We then performed Spearman correlation analysis on these top 50 genera between mothers and children. After FDR multiple comparison correction, 15 genera showed significant positive correlations (*P* < 0.05), including Faecalibacterium (*ρ* = 0.518, *P* = 0.002), Subdoligranulum (*ρ* = 0.462, *P* = 0.005), and Roseburia hominis (*ρ* = 0.458, *P* = 0.006) (Table 2). This suggests that these 15 genera may represent potential mother-child shared microbial taxa that warrant further strain level investigation.

**Table 2:** Spearman correlation analysis of the top 50 dominant genera shared between ASD children and their mothers.

genera	*ρ*	FDR-adjusted P	
Bifidobacterium	0.412	0.03	*
Escherichia-Shigella	0.386	0.021	*
Blautia	0.351	0.001	**
Eubacterium_hallii_group	0.435	0.002	**
Subdoligranulum	0.462	0.005	**
Faecalibacterium	0.518	0.002	**
Fusicatenibacter	0.372	0.008	**
Monoglobus	0.394	0.011	*
Roseburia	0.447	0.015	*
Intestinibacter	0.366	0.019	*
Butyricicoccus	0.381	0.023	*
Lactobacillus	0.429	0.028	*
Klebsiella	0.403	0.033	*
Roseburia_hominis	0.458	0.006	**
Enterococcus	0.347	0.045	*

ρ, Spearman correlation coefficient.

*P < 0.05.

**P < 0.01.

### 3.4 Association analysis of SCFA levels between ASD children and their mothers

**Figure 3:**
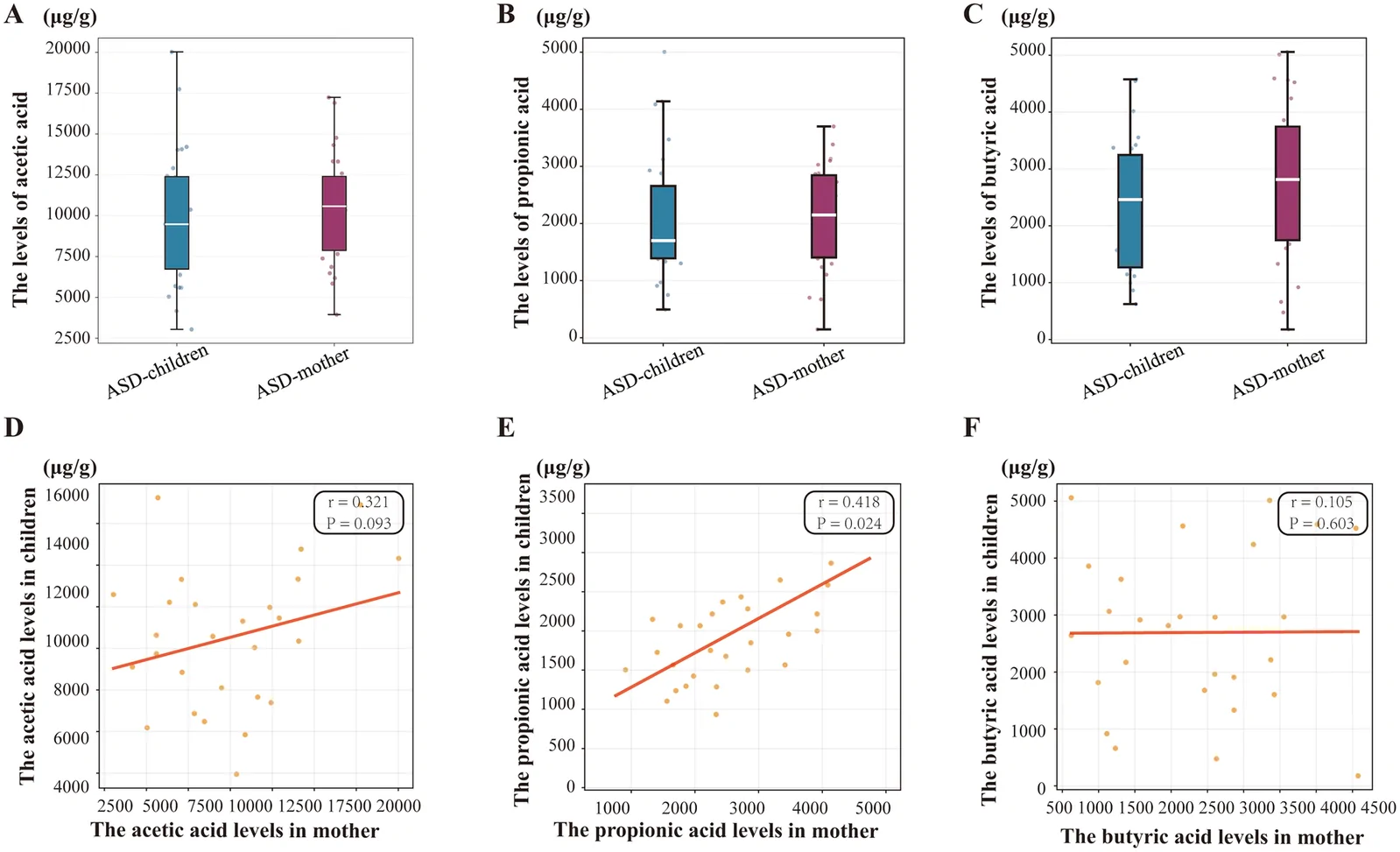
Comparison and correlation of SCFA levels between ASD children and their mothers. **(A–C)** Boxplots showing the differences in SCFA levels between children and mothers; **(D–F)** Scatter plots illustrating the correlations of SCFA levels between mother–child pairs.

To explore the relationship between the fatty acid profile of ASD children and their mothers, we analyzed the levels of acetic acid, propionic acid, and butyric acid in mother-child pairs using Microsoft Excel. The results showed that the average levels in children were 11,326.58 ± 572.6 μg/g for acetic acid, 2,021.55 ± 532.87 μg/g for propionic acid, and 3,023.15 ± 438.3μg/g for butyric acid. The average levels in mothers were 11,425.25 ± 898.0 μg/g for acetic acid, 1,965.04 ± 209.21 μg/g for propionic acid, and 2,516.02 ± 324.7 μg/g for butyric acid. The boxplot (Figures 3A–C) illustrated the differences in these three SCFA levels between mothers and children. Correlation analysis of SCFA levels between mothers and children (Figures 3D–F) revealed a moderately strong significant positive correlation for propionic acid (*ρ* = 0.418, *P* = 0.024), with children having slightly higher average levels than mothers (child/mother ratio ≈ 1.03). For acetic acid, a weak positive correlation was observed (*ρ* = 0.321), with a *P* value approaching significance (*P* = 0.093), and the average levels were roughly equal between mothers and children. For butyric acid, a weak positive correlation (*ρ* = 0.105) was not statistically significant (*P* = 0.603), although children had higher average levels than mothers (ratio ≈ 1.20). These results indicate a significant positive correlation in fecal propionic acid levels between ASD children and their mothers, while the correlations for acetic acid and butyric acid did not reach statistical significance.

### 3.5 Correlation analysis of key bacterial genera and SCFAs with CARS score in children with ASD

**Figure 4:**
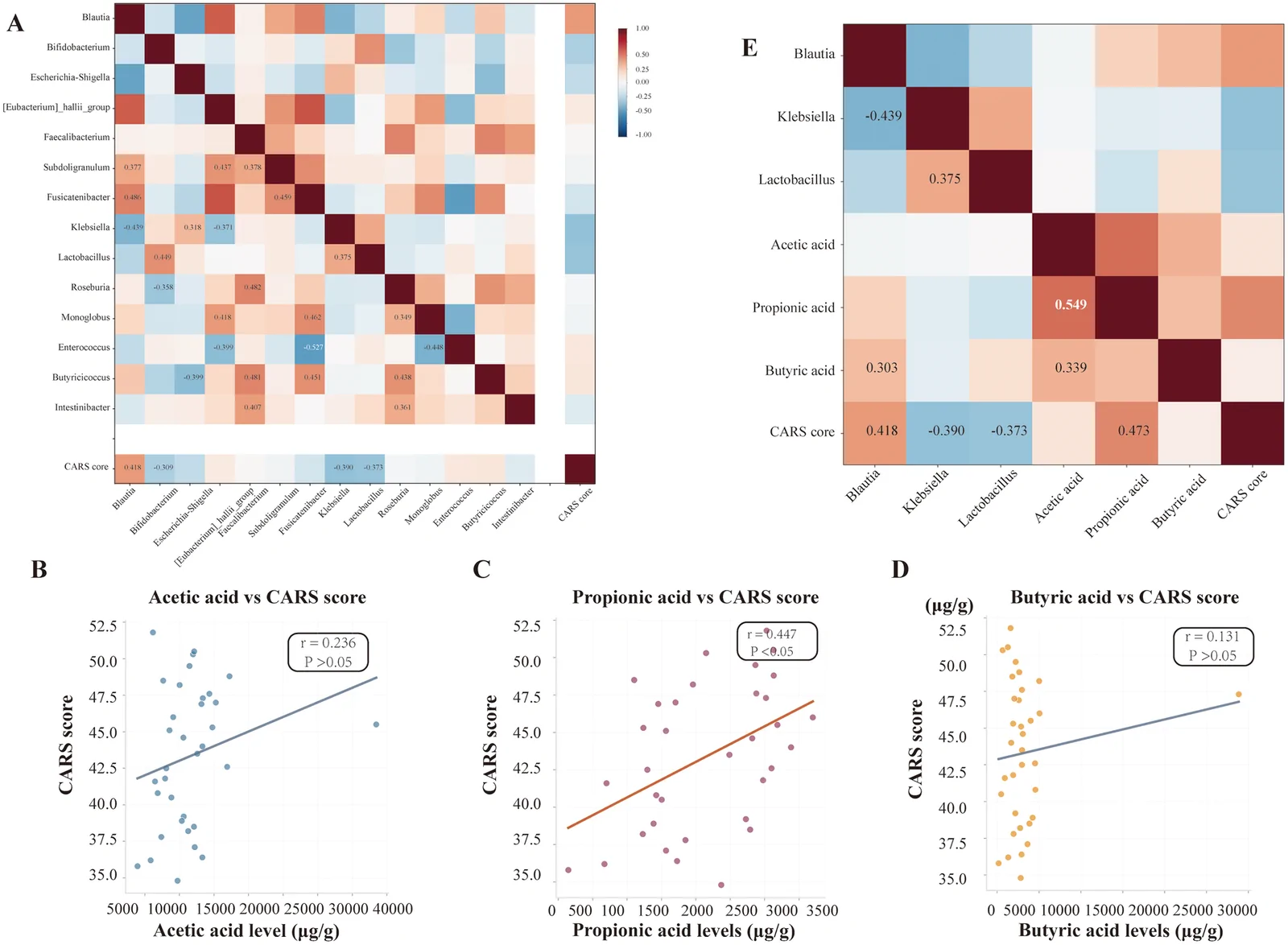
Correlation analysis of key bacterial genera and SCFAs with CARS scores in children with ASD. **(A)** Spearman correlation analysis between the relative abundance of 15 key bacterial genera (shared between mother and child) and CARS scores in children with ASD; **(B–D)** Spearman correlation analysis between fecal SCFA levels (acetic acid, propionic acid, and butyric acid) and CARS scores in children with ASD. **(E)** The interrelationships heat map among bacterial genera, SCFAs, and CARS scores.

Based on the above analysis, we obtained 15 key genera shared between mother and child, as well as three major SCFA (acetic acid, propionic acid, and butyric acid). To further evaluate the association between these indicators and ASD symptom severity, we first analyzed the Spearman correlation between the abundance of the 15 key bacterial genera in children with ASD and their CARS scores. The results showed that Blautia abundance was significantly positively correlated with CARS scores (*ρ*= 0.418, *P* = 0.017), while Klebsiella (*ρ* = −0.390, *P* = 0.027) and Lactobacillus (*ρ*= −0.373, *P* = 0.036) abundances were significantly negatively correlated with CARS scores. Bifidobacterium showed a weak negative correlation that did not reach statistical significance (*ρ* = −0.309, *P* = 0.085) (Figure 4A). Subsequently, we analyzed the correlation between fecal levels of acetic acid, propionic acid, and butyric acid in children and their CARS scores. The results showed that propionic acid level was significantly positively correlated with CARS scores (*ρ* = 0.473, *P* = 0.006), while the correlations of acetic acid (*ρ* = 0.119, *P* = 0.516) and butyric acid (*ρ*= 0.064, *P* = 0.727) with CARS scores did not reach statistical significance (Figures 4B–D). Further analysis of the associations among bacterial genera, SCFAs, and CARS scores (Figure 4E) revealed that CARS scores were positively correlated with Blautia (*ρ* = 0.418) and propionic acid (*ρ* = 0.473), suggesting a potential interrelationship among Blautia, propionic acid, and CARS.

### 3.6 Stratified analysis based on clinical and demographic indicators

**Figure 5:**
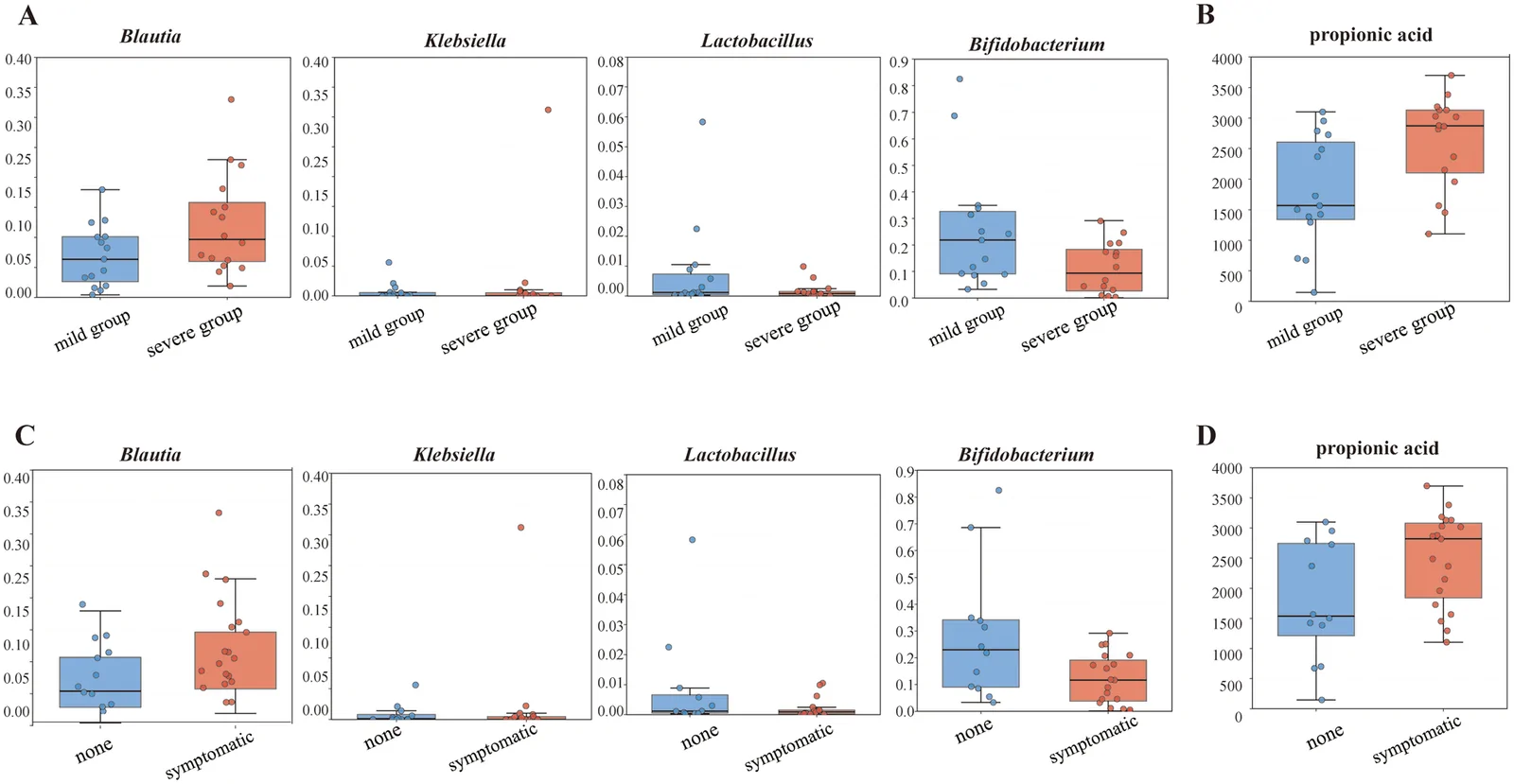
Stratified analysis of key bacterial genera and propionic acid levels based on CARS scores and gastrointestinal symptoms in children with ASD. **(A)** Comparison of Bifidobacterium, Blautia, Klebsiella, and Lactobacillus abundances between the mild-to-moderate and severe groups; **(B)** Comparison of fecal propionic acid levels between the mild-to-moderate and severe groups; **(C)** Comparison of Blautia, Bifidobacterium, Lactobacillus, and Klebsiella abundances between the symptomatic and asymptomatic groups; **(D)** Comparison of fecal propionic acid levels between the symptomatic and asymptomatic groups.

To explore the clinical heterogeneity of ASD, we divided the 37 children with ASD into a mild-to-moderate group (CARS scores < 37, *n* = 16) and a severe group (CARS scores ≥ 37, *n* = 21) according to the standardized CARS cut-off scores. Differences in the abundance of key bacterial genera and propionic acid levels were compared between the two groups. The results showed that the abundance of Bifidobacterium in the mild-to-moderate group was significantly higher than that in the severe group (*P* < 0.05). The abundances of Blautia and Klebsiella were slightly higher in the severe group than in the mild-to-moderate group, while the abundance of Lactobacillus was higher in the mild-to-moderate group than in the severe group; however, none of these differences reached statistical significance (Figure 5A). At the metabolite level, the mean propionic acid level in the severe group was significantly higher than that in the mild-to-moderate group (*P* < 0.05) (Figure 5B).

GI symptoms were assessed using a self-designed clinical symptom checklist (Supplementary Table S5). The checklist included diarrhea, constipation, abdominal distension, and abdominal pain. The presence of GI symptoms was defined as at least one of these four symptoms persisting for more than 3 months. Based on the presence or absence of at least one of the above symptoms, children with ASD were divided into a symptomatic group (*n* = 22) and an asymptomatic group (*n* = 15). Differences in the abundance of key bacterial genera and propionic acid levels were compared between the two groups. The results showed that the abundance of Blautia in the symptomatic group was higher than that in the asymptomatic group, with an increasing trend in bacterial abundance associated with gastrointestinal symptoms. The abundance of Bifidobacterium in the asymptomatic group was significantly higher than that in the symptomatic group. No significant differences were observed in the overall distributions of Lactobacillus and Klebsiella between the two groups (Figure 5C). The propionic acid level in the symptomatic group was higher than that in the asymptomatic group. These results suggest that propionic acid may be more closely associated with the severity of ASD symptoms (Figure 5D).

To evaluate the impact of delivery mode on mother-child gut microbiota sharing, we stratified the 37 mother–child pairs into vaginal delivery group (*n* = 16) and Cesarean section group (*n* = 21) based on delivery method. Four metrics were calculated to quantify the degree of microbiota sharing between each mother–child pair: shared ASV count, Ratio_child (shared ASV/child total ASV), Ratio_mother (shared ASV/mother total ASV), and Jaccard similarity index. Intergroup comparisons were performed using the Mann–Whitney U test. As summarized in Table 3, the vaginal delivery group exhibited significantly higher values across all four metrics compared to the Cesarean section group. Specifically, the vaginal delivery group had a mean shared ASV count of 18.31 ± 6.42, significantly higher than that of the Cesarean section group (12.76 ± 5.18, U = 92.5, *P* = 0.0047). The Ratio_child was 0.224 ± 0.076 in the vaginal delivery group versus 0.153 ± 0.063 in the Cesarean section group (U = 86.0, *P* = 0.0032); the Ratio_mother was 0.167 ± 0.061 versus 0.114 ± 0.048, respectively (U = 97.0, *P* = 0.0063). The Jaccard similarity index in the vaginal delivery group (0.098 ± 0.034) was also significantly higher than that in the Cesarean section group (0.064 ± 0.027, U = 81.5, *P* = 0.0021). These results demonstrated that mother–child pairs with vaginal delivery exhibited significantly greater gut microbiota sharing than those with Cesarean section.

**Table 3:** Delivery mode-stratified analysis of mother–child gut microbiota sharing.

Metric	Vaginal delivery (*n* = 16)	Cesarean section (*n* = 21)	U value	*P* value
Shared ASV count	18.31 ± 6.42	12.76 ± 5.18	92.5	0.0047
Ratio_child (Shared/Child total ASV)	0.224 ± 0.076	0.153 ± 0.063	86.0	0.0032
Ratio_mother (Shared/Mother total ASV)	0.167 ± 0.061	0.114 ± 0.048	97.0	0.0063
Jaccard similarity index	0.098 ± 0.034	0.064 ± 0.027	81.5	0.0021

### 3.7 External validation of key genera in public datasets

To independently validate the ASD-associated alterations of the 15 targeted genera identified in our study, we performed external differential abundance analyses using two publicly available gut metagenomic datasets (PRJNA453621 and PRJNA1018910). In the PRJNA453621 cohort, among the 15 targeted genera, four showed statistically significant differential abundance between ASD and control groups. Specifically, Bifidobacterium (LDA = 3.08, P.adj = 1.24 × 10⁻⁵), Escherichia-Shigella (LDA = 2.67, P.adj = 2.55 × 10⁻^3^), Intestinibacter (LDA = 2.24, P.adj = 8.49 × 10⁻^3^), and Lactobacillus (LDA = 2.15, P.adj = 1.18 × 10⁻^2^) were all significantly enriched in the ASD group. The remaining targeted genera, including Blautia, Eubacterium_hallii_group, Subdoligranulum, Faecalibacterium, Fusicatenibacter, Monoglobus, Roseburia, Butyricicoccus, Klebsiella, Roseburia_hominis, and Enterococcus, did not reach statistical significance or were not detected in this dataset (P.adj > 0.05). In the PRJNA1018910 cohort, seven were identified as differentially abundant. The genera Roseburia (LDA = 3.95, P.adj = 5.86 × 10⁻⁵), Fusicatenibacter (LDA = 3.61, P.adj = 5.40 × 10⁻⁴), Eubacterium_hallii_group (LDA = 3.50, P.adj = 1.88 × 10⁻^3^), Intestinibacter (LDA = 3.28, P.adj = 1.91 × 10⁻^2^), and Enterococcus (LDA = 3.25, P.adj = 2.54 × 10⁻^2^) were significantly enriched in the ASD group. In contrast, Lactobacillus (LDA = 3.39, P.adj = 1.51 × 10⁻^3^) and Klebsiella (LDA = 3.43, P.adj = 1.60 × 10⁻⁴) were significantly enriched in the control group. Notably, the direction of Lactobacillus enrichment was reversed compared to that observed in PRJNA453621 (where it was ASD-enriched), suggesting potential cohort-specific heterogeneity. The other targeted genera, including Bifidobacterium, Escherichia-Shigella, Blautia, Subdoligranulum, Faecalibacterium, Monoglobus, Butyricicoccus, and Roseburia_hominis, were not significantly different in this dataset. Notably, Intestinibacter was the only genus that demonstrated consistent ASD-enriched abundance across both external validation datasets (PRJNA453621: LDA = 2.24, P.adj < 0.01; PRJNA1018910: LDA = 3.28, P.adj < 0.05), highlighting its potential as a robust ASD-associated microbial marker (Table 4).

**Table 4:** External validation of the 15 targeted genera in two public datasets (PRJNA453621 and PRJNA1018910).

Target genus	PRJNA453621	PRJNA1018910	Consistency
Bifidobacterium	ASD (LDA = 3.08, P.adj = 1.24 × 10⁻⁵)	NS/ND	No
Escherichia-Shigella	ASD (LDA = 2.67, P.adj = 2.55 × 10⁻^3^)	NS/ND	No
Blautia	NS/ND	NS/ND	Yes (NS)
Eubacterium_hallii_group	NS/ND	ASD (LDA = 3.50, P.adj = 1.88 × 10⁻^3^)	
Subdoligranulum	NS/ND	NS/ND	Yes (NS)
Faecalibacterium	NS/ND	NS/ND	Yes (NS)
Fusicatenibacter	NS/ND	ASD (LDA = 3.61, P.adj = 5.40 × 10⁻⁴)	No
Monoglobus	NS/ND	NS/ND	Yes (NS)
Roseburia	NS/ND	ASD (LDA = 3.95, P.adj = 5.86 × 10⁻⁵)	No
Intestinibacter	ASD (LDA = 2.24, P.adj = 8.49 × 10⁻^3^)	ASD (LDA = 3.28, P.adj = 1.91 × 10⁻^2^)	Yes (ASD)
Butyricicoccus	NS/ND	NS/ND	Yes (NS)
Lactobacillus	ASD (LDA = 2.15, P.adj = 1.18 × 10⁻^2^)	Control (LDA = 3.39, P.adj = 1.51 × 10⁻^3^)	No (Reversed)
Klebsiella	NS/ND	Control (LDA = 3.43, P.adj = 1.60 × 10⁻⁴)	No
Roseburia_hominis	NS/ND	NS/ND	Yes (NS)
Enterococcus	NS/ND	ASD (LDA = 3.25, P.adj = 2.54 × 10⁻^2^)	No

## Discussion

ASD is a highly heterogeneous neurodevelopmental disorder. Both genetic and environmental factors play important roles in its pathogenesis. Maternal gut dysbiosis has emerged as a key environmental exposure, and accumulating evidence indicates that it can regulate fetal neurodevelopment through metabolite-mediated pathways, immune activation, and epigenetic reprogramming, thereby increasing ASD risk in offspring (20). Maternal immune activation (MIA) has also been implicated in ASD, with MIA-exposed offspring exhibiting gut dysbiosis and neurodevelopmental abnormalities (23, 24). These findings highlight the importance of maternal factors in ASD pathogenesis. In this study, we first evaluated gut microbiota sharing between ASD children and their mothers. Alpha diversity and PCoA analysis revealed no significant differences in overall microbial community composition between mothers and children, and the observed mother–child ASV sharing significantly exceeded random expectation (*P* < 0.001), indicating moderate gut microbiota overlap. Furthermore, vaginal delivery was associated with significantly greater sharing across all four metrics compared to Cesarean section, consistent with the established role of vertical transmission during birth canal exposure (25, 26). However, the persistence of shared ASVs in the Cesarean section group suggests that postnatal environmental factors (e.g., feeding, household contacts) also contribute to mother–child microbiota similarity (27, 28).

We identified 15 genera with significant positive correlations between mothers and children, including Faecalibacterium, Subdoligranulum, and Roseburia hominis, all butyrate-producing Firmicutes (29, 30). These findings align with Chen et al. (20), who reported that mother–child shared gut bacteria were associated with children's developmental levels and social deficits in ASD. We also observed a significant positive correlation for propionic acid between mothers and children, suggesting potential mother-to-child transmission of this SCFA. Regarding clinical severity, Blautia abundance positively correlated with CARS scores, while Klebsiella and Lactobacillus showed negative correlations. The Blautia-CARS association is consistent with previous reports (17, 31). Lactobacillus, a GABA-producing beneficial genus (32), showed the expected protective association. In contrast, our finding that Klebsiella negatively correlated with CARS scores is at odds with most previous studies showing Klebsiella enrichment in ASD (33–35). This discrepancy may reflect clinical heterogeneity or strain-level functional diversity. Bifidobacterium showed a non-significant negative trend consistent with its expected beneficial role (36). Propionic acid, a fermentation product of ASD-associated bacteria (37) was positively correlated with CARS scores, consistent with animal models where propionic acid induces ASD-like behaviors (38–40). This supports the involvement of propionic acid in ASD pathophysiology via the gut-brain axis.

Clinical stratification revealed that propionic acid levels were significantly higher in the severe versus mild-to-moderate group, and tended to be higher in children with versus without gastrointestinal symptoms. Bifidobacterium was more abundant in the milder and asymptomatic groups, while Blautia, Klebsiella, and Lactobacillus showed no significant between-group differences. These findings suggest that propionic acid may be a more robust indicator for clinical stratification than individual bacterial genera. Our observations are supported by studies showing that gastrointestinal symptoms correlate with CARS scores and that gut microbiota deviations in ASD are linked to symptom severity (41). External validation using two public datasets identified Intestinibacter as the only genus consistently enriched in ASD across both cohorts, suggesting it may be a robust ASD-associated microbial marker. However, inconsistent findings for other genera (e.g., reversed Lactobacillus enrichment between datasets) highlight the substantial cohort heterogeneity in ASD microbiome research.

Several limitations should be noted. The small sample size (37 dyads) limits statistical power, and the cross-sectional design precludes causal inference. Additionally, 16S rRNA sequencing provides limited species-level resolution, and the absence of a healthy control group prevents direct assessment of whether the observed mother-child sharing patterns are ASD-specific. Moreover, diet is known to be a critical factor influencing gut microbiota composition, and food selectivity is particularly prominent in children with ASD. However, detailed dietary assessments (e.g., 24-hour dietary recalls or food frequency questionnaires) were not collected in the present study. Therefore, we cannot exclude the potential confounding effects of different dietary patterns on the observed mother-child microbiota associations. Future studies should employ metagenomic sequencing, absolute quantification, and include healthy mother-child dyads for comparison, along with rigorous dietary records, to further clarify the specificity of ASD-related gut microbiota features. Additionally, GI symptoms were assessed using a self-designed checklist rather than a validated standardized questionnaire. This may have introduced information bias and limited the comparability of our findings with other studies. Future research should employ validated instruments for GI symptom assessment.

## Conclusion

In conclusion, this study systematically reveals shared gut microbiota and SCFA features between ASD children and their mothers, identifies 15 key genera significantly correlated between mother and child, and confirms a significant association between propionic acid and ASD symptom severity. External validation further suggests Intestinibacter as a potential robust ASD marker. Our findings provide new evidence for the role of mother-to-child vertical transmission in ASD and offer a theoretical basis for future propionic acid-targeted and probiotic-based interventions.

## Supplementary method. External validation procedures using public datasets

### Data sources and retrieval

We downloaded two public datasets from the NCBI Sequence Read Archive (SRA) under accession numbers PRJNA453621 and PRJNA1018910. PRJNA453621 contains fecal 16S rRNA gene sequencing data from ASD children and healthy controls; PRJNA1018910 contains fecal metagenomic data from ASD children and healthy controls. Both datasets are derived from child case-control populations rather than mother–child dyads.

### Data download and quality control

Raw sequencing data were downloaded using SRA Toolkit (v3.0.0) with the fastq-dump command and -split-files parameter to obtain paired-end reads. Quality assessment of raw reads was performed using FastQC (v0.11.9). Low-quality bases were trimmed using Trimmomatic (v0.39) with the following parameters: LEADING:3, TRAILING:3, SLIDINGWINDOW:4:20, MINLEN:50. High-quality reads after trimming were used for subsequent analyses.

### Sequence processing and taxonomic annotation

For the 16S rRNA dataset PRJNA453621, analysis was performed using QIIME 2 (2023.2). Denoising, chimera removal, and ASV (amplicon sequence variant) inference were conducted using qiime dada2 denoise-paired. Taxonomic assignment was performed using qiime feature-classifier classify-sklearn against the SILVA database (v138.2, 99% OTU reference sequences). For the metagenomic dataset PRJNA1018910.

### Differential abundance statistical analysis

Wilcoxon rank-sum test (Mann–Whitney U test) was used to compare relative abundances of Genera between ASD and healthy control groups. All statistical analyses were performed using R software (v4.2.1), with two-sided *P* < 0.05 considered statistically significant.

### Statement of analytical purpose

It should be emphasized that this external validation was designed solely to assess the reproducibility of genus-ASD phenotype associations at the independent population level, not to directly evaluate mother-child sharing rates. Since both public datasets are case-control designs without maternal samples, this analysis cannot address whether sharing rates are ASD-specific, a limitation that has been acknowledged in the main text Discussion.

## Data Availability

The data presented in this study are deposited in the NCBI BioProject database under accession number PRJNA1472580, and are directly accessible via the following link: https://www.ncbi.nlm.nih.gov/bioproject/PRJNA1472580.
